# Early Screening for the Parkinson Variant of Multiple System Atrophy: A 6‐Item Score

**DOI:** 10.1002/mdc3.14048

**Published:** 2024-04-24

**Authors:** Alessandra Fanciulli, Iva Stankovic, Omer Avraham, Milica Jecmenica Lukic, Adi Ezra, Fabian Leys, Georg Goebel, Florian Krismer, Igor Petrovic, Marina Svetel, Klaus Seppi, Vladimir Kostic, Nir Giladi, Werner Poewe, Gregor K. Wenning, Tanya Gurevich

**Affiliations:** ^1^ Department of Neurology Medical University of Innsbruck Innsbruck Austria; ^2^ Neurology Clinic, University Clinical Center of Serbia Belgrade Serbia; ^3^ Faculty of Medicine University of Belgrade Belgrade Serbia; ^4^ Movement Disorders Unit Neurological Institute, Tel‐Aviv Medical Center Tel‐Aviv Israel; ^5^ School of Medicine, Sagol School of Neuroscience Tel‐Aviv University Tel‐Aviv Israel; ^6^ Institute of Medical Statistics and Informatics Medical University of Innsbruck Innsbruck Austria; ^7^ Department of Neurology Provincial Hospital of Kufstein Kufstein Austria; ^8^ Sagol School of Neuroscience Tel‐Aviv University Tel‐Aviv Israel

**Keywords:** multiple system atrophy, Parkinson's disease, orthostatic hypotension, bladder dysfunction, postural instability

## Abstract

**Background:**

A 4‐item score based on ≥2 features out of orthostatic hypotension, overactive bladder, urinary retention and postural instability was previously shown to early distinguish the Parkinson‐variant of multiple system atrophy (MSA‐P) from Parkinson's disease (PD) with 78% sensitivity and 86% specificity.

**Objectives:**

To replicate and improve the 4‐item MSA‐P score.

**Methods:**

We retrospectively studied 161 patients with early parkinsonism [ie, ≤2 years disease duration or no postural instability, aged 64 (57; 68) years, 44% females] and a diagnosis of clinically established MSA‐P (n = 38) or PD (n = 123) after ≥24 months follow‐up.

**Results:**

The 4‐item MSA‐P score had a 92% sensitivity and 78% specificity for a final MSA‐P diagnosis. By including dopaminergic responsiveness and postural deformities into a 6‐item score (range: 0–6), reaching ≥3 points at early disease identified MSA‐P patients with 89% sensitivity and 98% specificity.

**Conclusions:**

The 6‐item MSA‐P score is a cost‐effective tool to pinpoint individuals with early‐stage MSA‐P.

Distinguishing the Parkinson‐variant of multiple system atrophy (MSA‐P) from Parkinson's disease (PD) is often difficult at early stages due to overlapping clinical features.^1^ Such suboptimal diagnostic accuracy is a major hurdle for patient's counseling and disease‐modifying trials targeting early disease phases.^2^

Clinicopathological studies consistently reported that postural instability and autonomic failure emerge earlier in MSA‐P than in PD.^3^, ^4^ In a recent study by the Innsbruck group, the combination at early disease of two or more clinical features including orthostatic hypotension (OH), symptoms of overactive bladder, urinary retention and postural instability into a 4‐item MSA‐P score indeed yielded a balanced sensitivity (78%) and specificity (86%) for a final MSA‐P diagnosis.^5^

Additional clinical MSA red flags were also shown to differentiate MSA from PD after an average disease duration of 5 years and may further increase the diagnostic accuracy for MSA‐P earlier in the disease course.^6^

In the present cooperation between the Tel Aviv Neurological Institute, the Belgrade Neurology Clinic, and the Innsbruck Department of Neurology, we aimed at:

1. Replicating the 4‐item MSA‐P score in an independent cohort of patients with recent onset of parkinsonism;
2. Verifying whether an expanded version of the MSA‐P score entailing additional clinical MSA red flags and information on dopaminergic responsiveness increases the clinical diagnostic accuracy for early MSA‐P.

## Methods

### Study Population

The medical records of patients with parkinsonism who had undergone a head‐up tilt or standing test at the Tel Aviv or Belgrade site between 2009 and 2021 were screened.

Patients were retrospectively included, if fulfilling the following criteria:

1. Completion of at least 3 min of head‐up tilt or standing test at *early disease*, ie, within 2 years from motor onset OR at a Hoehn & Yahr (H&Y) stage <3;
2. Diagnosis of clinically established MSA‐P^7^ or PD^8^ at last‐available neurological follow‐up;
3. Available follow‐up time of ≥24 months from the timepoint of the head‐up tilt or standing test.

Exclusion criteria were:

1. Diagnosis of dementia, other major neurologic or psychiatric diseases at the time of head‐up tilt or standing test;
2. Incomplete medical information available;
3. Poor head‐up tilt or standing test bio‐signal quality.

### Clinical‐Demographic Data‐Set

For each patient, a local study team member extracted the following information from the medical records contemporary to the head‐up tilt or standing test:

1. Clinical‐demographic characteristics (age, sex, disease duration from the beginning of motor symptoms, age at disease onset, H&Y stage);
2. Medication schedule (number of drugs/day, L‐Dopa equivalent daily dose,^9^ anti‐hypotensive or anti‐hypertensive medication intake);
3. Presence of diabetes or other cardiovascular comorbidities (ie, hypertension, ischemic, arrhythmic or structural heart disease);
4. Documented postural instability within 2 years from motor onset^10^, ^11^;
5. Symptoms of overactive bladder (urinary urge, incontinence or use of appliances), urinary voiding difficulties (based on medical history or post‐void residual urine volume >100 mL,^12^ whenever available), postural deformities (including Pisa syndrome, disproportionate antecollis, contractures of the hands or feet),^6^ bulbar dysfunction (dysphonia, dysarthria or dysphagia),^6^ respiratory symptoms (stridor, inspiratory sighs)^6^ or emotional incontinence^6^;
6. Dopaminergic responsiveness, ie, good, poor or undetermined, as defined in the former Innsbruck study.^5^

### Head‐Up Tilt and Standing Test

Heart rate (HR), systolic and diastolic blood pressure (BP) values at the end of a 5‐min supine phase, and after 3 min in the upright position were extracted from the identified head‐up tilt and standing tests.^13^, ^14^, ^15^ OH was defined by a BP fall ≥20/10 mmHg within 3 min in the upright position.^16^

### Diagnostic Gold Standard

The diagnostic criteria for clinically established MSA^7^ and PD^8^ were retrospectively applied to the last available visit of each patient by experienced Tel Aviv or Belgrade investigators other than those collecting the clinical‐demographic information, and served as a clinical diagnostic gold standard.

### Statistical Analysis

Dichotomous variables were summarized by frequency (percentage) and compared with the *Χ* ^2^ or Fisher's exact test. Quantitative variables were tested for normality with the Kolmogorov–Smirnov test, reported by median (1st quartile; 3rd quartile) and compared using the *T*‐ or Mann–Whitney‐*U* test.

We first compared the pooled Tel Aviv and Belgrade MSA and PD cohorts with the original Innsbruck study population used to generate the 4‐item MSA‐P score.^5^ The clinical‐demographic characteristics of the newly recruited MSA versus PD cohorts were subsequently compared in a univariate fashion and the 4‐item MSA‐P score was calculated for each patient. The diagnostic accuracy of a cumulative score ≥2 at early disease (score range: 0–4) in identifying patients suffering from MSA‐P was subsequently weighed against the neurological diagnosis at last available follow‐up (sensitivity, specificity, positive and negative predictive value, area under the ROC curve).

In a second step, a decision‐tree algorithm for a final MSA‐P diagnosis was built with an observer‐independent CHAID methodology by adding additional MSA clinical red flags^6^ and information on dopaminergic responsiveness to the previously calculated 4‐item MSA‐P score (fixed first decisional node). The robustness of the model was verified with a cross validation technique.

The additionally identified characteristics, which early‐distinguished MSA‐P from PD were used to expand the MSA‐P score that was recalculated for each patient. A Youden‐index was applied to identify the score cut‐off with the highest diagnostic accuracy for a final MSA‐P diagnosis. This was again verified towards the final neurological diagnosis.

Statistical analysis was performed with IBM‐SPSS and Med Calc. Two‐tailed *P*‐values <0.05 were considered statistically significant. A Bonferroni correction was applied to multiple testing.

## Results

### Study Population

Thirty‐eight patients with MSA and 123 with PD were included in the present study. The clinical‐demographic characteristics of the study population are summarized in Table 1. A comparison with the previous Innsbruck cohort and between the Tel Aviv and Belgrade study cohorts is provided in Table S1 and S2.

**TABLE 1:** Clinical‐demographic characteristics of the pooled Tel Aviv and Belgrade study population.

Variable	PD	MSA‐P	*P*
n	123	38	‐
Age	64 (58; 68)	62 (56; 67)	0.431
Sex, female	53 (43)	18 (47)	0.642
Age at motor symptoms onset, years	61 (55; 65)	61 (54; 66)	0.881
Disease duration from motor symptoms onset, years	2 (1.8; 4)	2 (2;2)	0.006
Hoehn & Yahr stage	2 (1.5; 2)	3 (2.5; 4)	**<0.001**
Available follow‐up time, months	108 (53; 120)	38 (24; 48)	**<0.001**
Diabetes	13 (11)	2 (5)	0.524
Cardiovascular comorbidities	60 (49)	15 (40)	0.315
Total No. of drugs/day	3 (2; 5)	4 (2; 5)	0.292
L‐Dopa equivalent daily dose, mg/day	350 (263; 475)	388 (166; 500)	0.954
Use of anti‐hypotensive medications	0 (0)	3 (8)	0.012
Use of anti‐hypertensive medications	54 (44)	13 (34)	0.289
OH	19 (15)	30 (79)	**<0.001**
Symptoms of overactive bladder	67 (55)	32 (84)	**<0.001**
Urinary voiding difficulties	21 (17)	22 (58)	**<0.001**
Postural instability within 2 years from motor onset	2 (2)	28 (74)	**<0.001**
Dopaminergic responsiveness			**<0.001**
Poor	0 (0)	24 (63)	
Undetermined	11 (9)	9 (24)	
Good	112 (91)	5 (13)	
Postural deformities	1 (1)	11 (29)	**<0.001**
Pisa syndrome	0 (0)	6 (16)	**<0.001**
Antecollis	0 (0)	4 (11)	0.003
Contractures of hands or feet	1 (1)	3 (8)	0.041
Bulbar dysfunction	18 (15)	25 (66)	**<0.001**
Dysarthria	12 (10)	17 (44)	**<0.001**
Dysphagia	8 (7)	14 (37)	**<0.001**
Respiratory symptoms	2 (2)	19 (50)	**<0.001**
Stridor	2 (2)^a^	8 (21)	**<0.001**
Inspiratory sighs	0 (0)	12 (32)	**<0.001**
Emotional incontinence	0 (0)	3 (8)	0.012
4‐item MSA‐P score			**<0.001**
0 points	41 (33)	0 (0)	
1 point	55 (45)	3 (8)	
2 points	27 (22)	9 (24)	
3 points	0 (0)	13 (34)	
4 points	0 (0)	13 (34)	
4‐item MSA‐P score category			**<0.001**
Low‐risk (0–1 point)	96 (78)	3 (8)	
High‐risk (2–4 points)	27 (22)	35 (92)	

Significant *P* values after Bonferroni correction for multiple comparisons are marked in bold.

Compared to the 2019 Innsbruck PD cohort^5^ (n = 159), the newly recruited Tel Aviv—Belgrade PD cohort was significantly younger and on a lower number of medications per day, had a shorter disease duration at the time of head‐up tilt or standing test and a longer follow‐up time available (Table S1).

Compared to the 2019 Innsbruck MSA‐P patients^5^ (n = 27), the present MSA‐P cohort showed comparable clinical‐demographic characteristics, but had a longer follow‐up time available (Table S1).

When comparing the participants between the Tel Aviv and Belgrade sites, both the PD and MSA‐P study cohorts showed in‐between sites clinical heterogeneities, but no significant differences in their distribution into low versus high risk of suffering from MSA‐P according to the 4‐items MSA‐P score (Table S2).

^a^ To inquire about stridor, physicians demonstrated it to the patients and their partners with their own voice, asking if they have ever heard such sound. We acknowledge that the partners of these two Tel Aviv PD cases might have misinterpreted nocturnal stridor with snoring.

### Validation of the 4‐Item MSA‐P Score

In the newly recruited Tel Aviv‐Belgrade cohort, 92% of the patients with a final MSA‐P and 22% of those with a final PD diagnosis were classified at high‐risk of suffering from MSA‐P at early disease. The sensitivity of the 4‐item MSA‐P score for a final MSA‐P diagnosis was 92% (95% confidence interval, c.i.:79–98%), the specificity 78% (95% c.i.:70–85%), the positive predictive value 57% (95% c.i.: 48–65%) and the negative predictive value 97% (95% c.i.:92–99%). The area under the ROC curve was 0.939 (95% c.i.:0.895–0.983).

### Expansion of the MSA‐P Score

**Figure 1:**
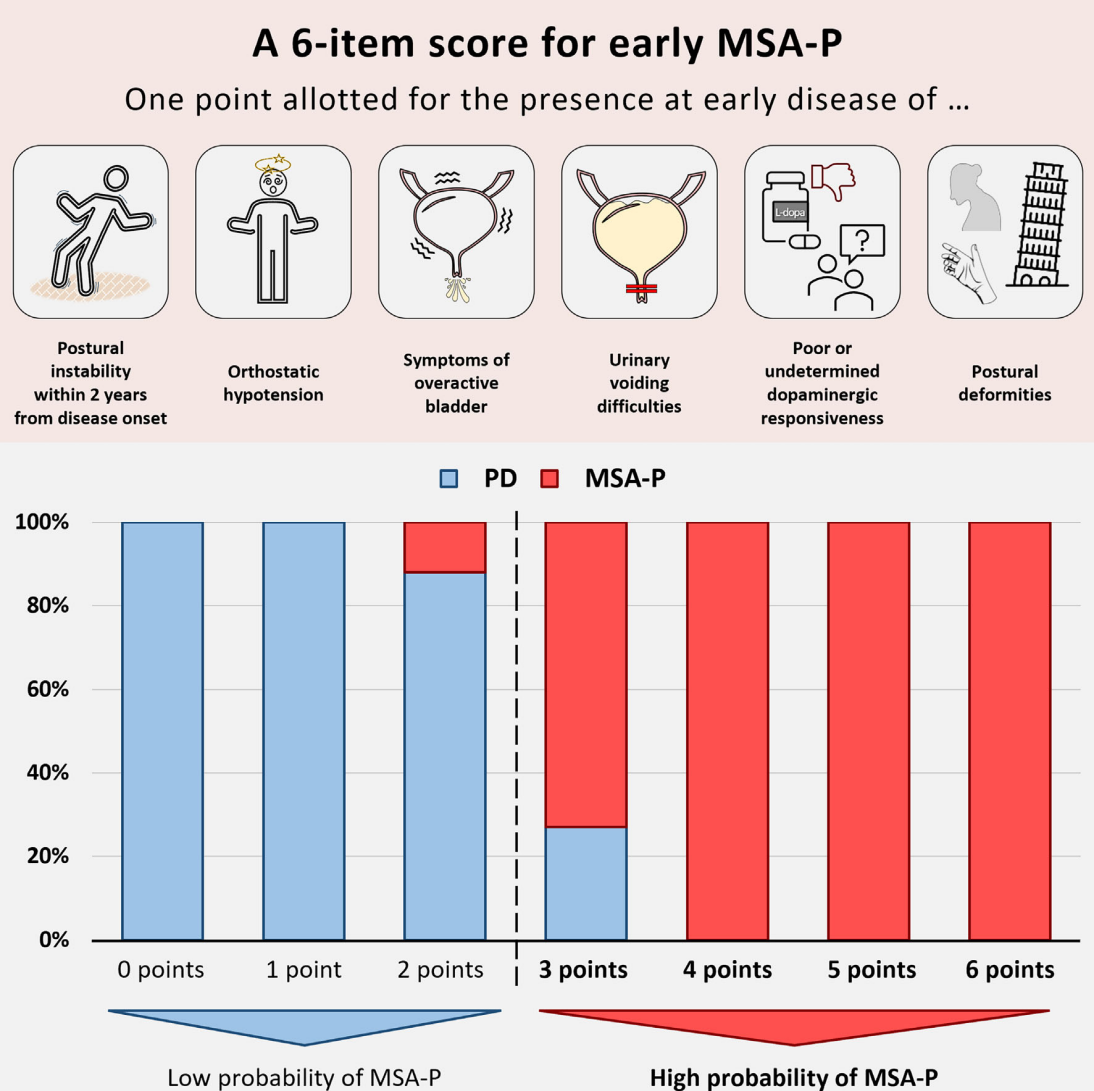
A 6‐item score for early distinguishing MSA‐P from PD. The upper panel shows the clinical features included in the 6‐item MSA‐P score. The lower panel shows the 6‐item MSA‐P score distribution in the pooled Tel Aviv‐Belgrade retrospective cohort of individuals with early parkinsonism (i.e., assessed within 2 years from motor onset or prior to developing postural instability) and a diagnosis of clinically established MSA‐P or PD after ≥24 months follow‐up. The Tel Aviv and Belgrade study cohorts did not show a significant difference in their distribution into low versus high risk of suffering from MSA‐P according to the 6‐items MSA‐P score (Table S2).

The decision‐tree algorithm identified poor or undetermined dopaminergic responsiveness and the presence of postural deformities as further clinical red flags early distinguishing MSA‐P from PD (Fig. S1). By increasing the MSA‐P score to 6 items, a cut‐off of ≥3 achieved the highest Youden‐index for discriminating individuals at low (0–2 points) versus high‐risk (3–6 points) of suffering from MSA‐P. The expanded 6‐item MSA‐P score had a sensitivity of 89% (95% c.i.:75–97%), a specificity of 98% (95% c.i.:93–99%), a positive predictive value of 92% (95% c.i.:79–97%) and a negative predictive value of 97% (95% c.i.:92–99%) for a final MSA‐P diagnosis. The AUC of the ROC curve was 0.935 (95% c.i.: 0.876–0.994, Fig. 1).

## Discussion

The 4‐item MSA‐P score was developed as a widely accessible tool to screen patients with early parkinsonism for their likelihood to suffer from MSA—a low MSA‐risk was defined by scores of 0 to 1, while scores of 2–4 points indicated a high‐risk. In the present validation cohort of patients from two independent centers, the sensitivity was higher compared to the original study (92% vs. 78%), while specificity was somewhat lower (78% vs. 86%), and the positive and negative predictive values for a final MSA‐P diagnosis were similar (57% vs. 50% and 97% vs. 96%, respectively). The increased sensitivity is likely due to the fact that an OH diagnosis based on a standing test^17^ and a history of urinary disturbances were sufficient to assign a point for the respective items of the MSA‐P score in the present study. Such methodological decision was consciously taken bearing in mind that access to autonomic function testing^18^ is often limited outside of MSA referral centers,^19^ yet bedside screening measures like the standing test provide significant diagnostic and therapeutic guidance when included in the work‐up of individuals with early parkinsonism. This same decision however also likely explains the lower specificity observed, since non‐neurogenic causes of OH and urinary disturbances such as polypharmacy, perineal laxity in women or prostate hypertrophy in men were not systematically excluded like in the previous study. Despite such lower rigorousness in assessing autonomic disturbances, which is probably similar to that encountered in the diagnostic work‐up of MSA in underserved geographical areas, the 4‐item MSA‐P score yielded an overall balanced accuracy for an early clinical distinction between MSA‐P and PD.

By including the pattern of dopaminergic responsiveness and development of postural deformities within 2 years from motor onset, the expanded 6‐item MSA‐P score gained specificity and positive predictive value compared to the original 4‐item score. Even though one‐third of MSA individuals may initially show some dopaminergic responsiveness,^1^, ^20^ this is in fact usually not as good as in PD and frequently associated with cranio‐cervical or limb dystonia, in the absence of PD‐typical limb dyskinesia.^21^ Postural deformities, such as antecollis and Pisa syndrome, also point against a PD diagnosis if developing early in the disease course.^8^, ^22^

Our study has limitations. The present cohort lacked a neuropathological diagnosis and a quantitative assessment of dopaminergic responsiveness. The latter was due to the retrospective study design that also potentially introduced a selection bias and hindered a standardized documentation of autonomic disturbances eventually preceding motor symptoms. There was however a prolonged follow‐up time of ≥24 months available, following which experienced neurologists carefully verified the neurological diagnosis against the latest PD and MSA diagnostic criteria.^7^, ^8^ The latter were recently shown to have modest sensitivity, but consistently high specificity for a MSA diagnosis in neuropathological studies.^23^, ^24^

In recent years, science is moving towards a biological definition of a‐synucleinopathies,^25^ which is key for enrolling affected individuals in trials with technologies targeting the disease‐specific mechanisms of the disease. We are however still far for the implementation of biological diagnostic criteria in clinical practice and individuals with MSA are often diagnosed 4 to 5 years into their disease course.^26^, ^27^

We conclude that a simple scoring system like the 6‐item MSA‐P score will aid selecting patients for studies of biomarker sensitivity and specificity, even in areas where autonomic function laboratories are not available.

## Author Roles

(1) Research project: A. Conception, B. Organization, C. Execution; (2) Statistical Analysis: A. Design, B. Execution, C. Review and Critique; (3) Manuscript: A. Writing of the First Draft, B. Review and Critique.

A.F.: 1A, 1B, 1C, 2A, 2B, 3A.

I.S.: 1B, 1C, 2C, 3B.

O.A.: 1C, 2C, 3B.

M.J.L.: 1C, 2C, 3B.

A.E.: 1C, 2C, 3B.

F.L.: 1C, 2C, 3B.

G.G.: 1C, 2C, 3B.

F.K.: 1C, 2C, 3B.

I.P.: 1C, 2C, 3B.

M.S.: 1C, 2C, 3B.

K.S.: 1C, 2C, 3B.

V.K.: 1C, 2C, 3B.

N.G.: 1A, 1C, 2C, 3B.

W.P.: 1A, 1C, 2C, 3B.

G.K.W.: 1A, 1C, 2A, 2C, 3B.

T.G.: 1A, 1B, 1C, 2A, 2C, 3B.

## Disclosures

**Ethical Compliance Statement:** The protocol was approved by the institutional Review Board of the Tel Aviv Medical Center (approval No. 0458‐11). Per Serbian law, no ethical approval or patient informed consent was required for retrospective studies conducted at the Faculty of Medicine of the University of Belgrade. We confirm that we have read the Journal's position on issues involved in ethical publication and affirm that this work is consistent with those guidelines.

**Funding Sources and Conflicts of Interest:** AF and FK are supported by the Austrian Science Fund (FG‐2700). The authors declare no relevant conflicts of interest related to the present work. The full financial disclosures of all authors are provided at the end of the manuscript.

**Financial Disclosures of All Authors (for the Preceding 12 Months)**: A.F.: Dr Fanciulli reports royalties from Springer Verlag, speaker fees and honoraria from Theravance Biopharma, GE Health Care, Broadview Ventures, Austrian Autonomic Society, Stopp‐HSP, Elsevier, International Parkinson Disease and Movement Disorders Society and research grants from the FWF‐Austrian Science Fund, Medical University of Innsbruck, US MSA Coalition, Dr Johannes and Hertha Tuba Foundation and Austrian Exchange Program, outside of the present work. I.S.: nothing to disclose. O.A.: nothing to disclose. M.J.L.: Dr Jecmenica Lukic reports speaker fees and honoraria from Salveo Pharma, International Parkinson Disease and Movement Disorders Society, outside of the present work. A.E.: nothing to disclose. F.L.: nothing to disclose. GG: nothing to disclose. F.K.: Dr Krismer reports personal fees from Takeda, Sanofi, Teva, Bial and Koneksa Health in the past 12 months and he has ongoing grant support paid to his institution from the Austrian Science Fund (FWF) and the National Institutes of Health, outside of the submitted work. I.P.: Dr Petrovic reports speaker honoraria from Salveo Pharma, Pharma Swiss, Makpharm and Inopharm, outside of the present work. M.S.: Dr Svetel reports speaker honoraria from Makpharm and Salveo Pharma, outside of the present work. K.S.: Dr Seppi reports personal fees from Ono Pharma UK Ltd, Teva, UCB Pharma, Lundbeck, grants, personal fees from AOP Orphan Pharmaceuticals AG, personal fees from Roche Pharma, Grünenthal, Stada, AbbVie, Ever Pharma, Licher Pharma, Biogen, BIAL, grants, personal fees from International Parkinson and Movement Disorders Society, grants from Michael J. Fox Foundation, grants from FWF Austrian Science Fund, outside the submitted work. V.K.: nothing to disclose. N.G.: Dr Giladi has no conflict of interest related to this work. He serves as consultant to Sionara, NeuroDerm, Pharma2B, Denali, Neuron23, Sanofi‐Genzyme, Biogen and Abbvie. He received royalties from Lysosomal Therapeutics (LTI) and payment for lectures at Abbvie, Sanofi‐Genzyme and Movement Disorder Society. He received research support from the Michael J. Fox Foundation, the National Parkinson Foundation, the European Union and the Israel Science Foundation as well as from Teva NNE program, Biogen and Ionis. He receives support from the Sieratzki Family Foundation and the Aufzien Academic Center in Tel Aviv University. W.P.: Dr Poewe reports consultancy and lecture fees from AC Immune, Alterity, Affiris, Biogen, Lundbeck, Takeda, in relation to clinical drug development programmes for MSA. G.K.W.: Dr Wenning reports consultancy and lecture fees from Inhibicase, Ono, and Theravance and research grants from the FWF‐Austrian Science Fund, US MSA Coalition, Parkinson Fonds Austria, Dr Johannes and Hertha Tuba Foundation, outside of the present work. T.G.: Dr Gurevich reports consultancy fees from Truemed, Abbvie and Tradis Gat, travel support for her and her team from Alphamedix, Abbvie and Medison and grants from International Parkinson Disease and Movement Disorders Society and Parkinson's Foundation, outside of the present work.

## Supporting information


**TABLE S1.** Clinical‐demographic characteristics of the 2019 Innsbruck versus the pooled Tel Aviv and Belgrade PD and MSA‐P study cohorts.
**TABLE S2.** Clinical‐demographic characteristics of the Tel Aviv versus Belgrade PD and MSA‐P study cohorts.
**Figure S1.** Observer‐independent CHAID regression tree algorithm for the expansion of the 4‐item MSA‐P score.

## Data Availability

I.S., M.J.L. and T.G. take responsibility for the integrity of data from the Belgrade and Tel Aviv site. A.F. takes responsibility for the accuracy of the data analysis. The authors agree to share any data not included in the present manuscript or its supplementary material upon reasonable request by any qualified investigator.
