# Supplementary material for: Early Screening for the Parkinson Variant of Multiple System Atrophy: A 6‐Item Score
Source: Mov Disord Clin Pract. 2024 Apr 24;11(7):867–73. doi: 10.1002/mdc3.14048 (PMC11233846; doi:10.1002/mdc3.14048)
Supplement: Supplementary file 1 — TABLE S1. Clinical‐demographic characteristics of the 2019 Innsbruck versus the pooled Tel Aviv and Belgrade PD and MSA‐P study cohorts. TABLE S2. Clinical‐demographic characteristics of the Tel Aviv versus Belgrade PD and MSA‐P study cohorts. Figure S1. Observer‐independent CHAID regression tree algorithm for the expansion of the 4‐item MSA‐P score. [file MDC3-11-867-s001.docx]

# Early screening for the Parkinson variant of multiple system atrophy: a 6-item score

***Supplementary material***

Alessandra Fanciulli^1^, MD, PhD, Iva Stankovic^2,3*^, MD, PhD, Omer Avraham^4,7*^, MD, Milica Jecmenica Lukic^2,3**^, MD, PhD, Adi Ezra^4**^, MHA, Fabian Leys^1^, MD, PhD, Georg Goebel^5^, PhD, Florian Krismer^1^, MD, PhD, Igor Petrovic^2,3^, MD, PhD, Marina Svetel^2,3^, MD, PhD, Klaus Seppi^6,1^, MD, Vladimir Kostic^2,3^, MD, PhD, Nir Giladi^4,7,8^, MD Werner Poewe^1^, MD, Gregor K. Wenning^1†^, MD, PhD, MSC, Tanya Gurevich^4,7,8^, MD.

*^1^Department of Neurology, Medical University of Innsbruck – Innsbruck, Austria
^2^Neurology Clinic, University Clinical Center of Serbia – Belgrade, Serbia
^3^Faculty of Medicine, University of Belgrade – Belgrade, Serbia
^4^Movement Disorders Unit, Neurological Institute, Tel-Aviv Medical Center – Tel-Aviv, Israel
^5^Institute of Medical Statistics and Informatics, Medical University of Innsbruck, Innsbruck – Austria
^6^Department of Neurology, Provincial Hospital of Kufstein – Kufstein, Austria
^7^ School of Medicine, Sagol School of Neuroscience, Tel-Aviv University – Tel-Aviv, Israel*

*^8^Sagol School of Neuroscience, Tel-Aviv University – Tel-Aviv, Israel*

*/** These authors contributed equally.

† Author deceased.

Supplementary Table 1 - Clinical-demographic characteristics of the 2019 Innsbruck versus the pooled Tel Aviv and Belgrade PD and MSA-P study cohorts

Dichotomous variables are summarized by frequency (percentage), quantitative variables by median (1^st^ quartile; 3^rd^ quartile). Significant p values after Bonferroni correction for multiple comparisons are marked in bold.

| **Variable** | **2019**  **Innsbruck PD** | **Tel Aviv – Belgrade**  **PD** | **p** | **2019**  **Innsbruck MSA-P** | **Tel Aviv – Belgrade**  **MSA-P** | **p** |
| --- | --- | --- | --- | --- | --- | --- |
| n | 159 | 123 | - | 27 | 38 | - |
| Age | 68 (62; 73) | 64 (58; 68) | **<0.001** | 67 (59; 72) | 62 (56; 67) | 0.131 |
| Sex, female | 109 (69) | 53 (43) | 0.044 | 18 (67) | 18 (47) | 0.258 |
| Age at disease onset, years | 63 (55; 69) | 61 (55; 65) | 0.068 | 64 (56; 70) | 61 (54; 66) | 0.171 |
| Disease duration from motor onset, years | 4 (2; 7) | 2 (1.8; 4) | **<0.001** | 1.5 (1; 2.5) | 2 (2;2) | 0.079 |
| Hohen & Yahr stage | 2 (2; 2.5) | 2 (1.5; 2) | **<0.001** | 2.5 (2;3) | 3 (2.5; 4) | 0.057 |
| Available follow-up time, months | 45 (22; 76) | 108 (53; 120) | **<0.001** | 22 (1; 34) | 38 (24; 48) | **<0.001** |
| Diabetes | 7 (4) | 13 (11) | 0.045 | 4 (15) | 2 (5) | 0.224 |
| Cardiovascular comorbidities | 67 (42) | 60 (49) | 0.266 | 13 (48) | 15 (40) | 0.486 |
| Total No. of drugs/day | 4 (3; 7) | 3 (2; 5) | **<0.001** | 7 (3; 8) | 4 (2; 5) | 0.003 |
| L-Dopa equivalent daily dose, mg/day | 450 (210; 792) | 350 (263; 475) | 0.041 | 550 (300; 798) | 388 (166; 500) | 0.046 |
| Use of anti-hypotensive medications | 5 (3) | 0 (0) | 0.07 | 2 (7) | 3 (8) | 1 000 |
| Use of anti-hypertensive medications | 54 (34) | 54 (44) | 0.089 | 10 (37) | 13 (34) | 0.814 |
| OH | 34 (21) | 19 (15) | 0.206 | 16 (59) | 30 (79) | 0.085 |
| Symptoms of overactive bladder  (n=151 PD for the Innsbruck site) | 64 (42) | 67 (55) | 0.046 | 22 (82) | 32 (84) | 0.772 |
| Urinary voiding difficulties (n=151 PD for the Innsbruck site) | 9 (6) | 21 (17) | 0.006 | 9 (33) | 22 (58) | 0.051 |
| Postural instability within 2 years from motor onset | 3 (2) | 2 (2) | 1.000 | 12 (44) | 28 (74) | 0.017 |
| Dopaminergic responsiveness* |  |  | 0.261 |  |  | 0.187 |
| Poor | 0 (0) | 0 (0) |  | 10 (42) | 24 (63) |  |
| Undetermined | 20 (14) | 11 (9) |  | 7 (29) | 9 (24) |  |
| Good | 118 (86) | 112 (91) |  | 7 (29) | 5 (13) |  |
| 4-items MSA-P score (n=151 PD for the Innsbruck site) |  |  | 0.196 |  |  | 0.065 |
| 0 points | 66 (44) | 41 (33) |  | 0 (0) | 0 (0) |  |
| 1 point | 64 (42) | 55 (45) |  | 6 (22) | 3 (8) |  |
| 2 points | 19 (13) | 27 (22) |  | 13 (48) | 9 (24) |  |
| 3 points | 2 (1) | 0 (0) |  | 5 (19) | 13 (34) |  |
| 4 points | 0 (0) | 0 (0) |  | 3 (11) | 13 (34) |  |
| 4-items MSA-P score category (n=151 PD for the Innsbruck site) |  |  | 0.08 |  |  | 0.102 |
| Low risk (i.e., 0-1 point) | 130 (86) | 96 (78) |  | 6 (22) | 3 (8) |  |
| High risk (i.e., ≥2 points) | 21 (14) | 27 (22) |  | 21 (78) | 35 (92) |  |

* Dopaminergic responsiveness was defined both in the 2019 and current study as follows:
*i. good*, if both the patient and treating physician agreed on a beneficial effect of dopaminergic medications;
*ii. poor*, if both agreed on the lack of benefit from dopaminergic treatment;
*iii. undetermined*, in case of disagreement between the patient and physician judgement, or whenever the observed changes were insufficient to conclude on efficacy (i.e., due to low daily dopaminergic dosage or reduced compliance because of side effects).

Supplementary Table 2 Clinical-demographic characteristics of the Tel Aviv versus Belgrade PD and MSA-P study cohorts

Dichotomous variables are summarized by frequency (percentage), quantitative variables by median (1^st^ quartile; 3^rd^ quartile). Significant p values after Bonferroni correction for multiple comparisons are marked in bold.

Both the PD and MSA-P study cohorts showed clinical heterogeneities between the Tel Aviv and Belgrade sites, but no significant in-between sites differences in their distribution into low versus high risk of suffering from MSA-P according to the 4-items and 6-items MSA-P score.

| **Variable** | **PD cohort** | |  | **MSA cohort** | |  |
| --- | --- | --- | --- | --- | --- | --- |
|  | **Tel Aviv**  **site** | **Belgrade**  **site** | **p** | **Tel Aviv**  **site** | **Belgrade site** | **p** |
| n | 34 | 89 | - | 17 | 21 | - |
| Age | 66 (61; 71) | 62 (57; 67) | 0.067 | 68 (64; 74) | 57 (53; 61) | **<0.001** |
| Sex, female | 11 (32) | 42 (47) | 0.137 | 8 (47) | 10 (48) | 0.973 |
| Age at disease onset, years | 63 (58; 66) | 60 (54; 64) | 0.250 | 66 (62; 73) | 55 (51; 60) | **<0.001** |
| Disease duration from motor onset, years | 2.5 (2; 6) | 2 (2; 3) | 0.171 | 2 (2; 2) | 2 (2; 2) | 0.416 |
| Hohen & Yahr stage | 2 (2; 2.5) | 2 (1; 2) | **<0.001** | 3 (3; 4) | 3 (2; 3) | 0.128 |
| Available follow-up time, months | 37 (30; 50) | 108 (105; 120) | **<0.001** | 41 (24; 51) | 36 (24; 48) | 0.609 |
| Diabetes | 7 (21) | 6 (7) | 0.025 | 2 (12) | 0 (0) | 0.193 |
| Cardiovascular comorbidities | 18 (53) | 42 (47) | 0.568 | 10 (59) | 5 (24) | 0.028 |
| Total No. of drugs/day | 7 (4; 8) | 2 (2; 3) | **<0.001** | 5 (4; 8) | 2 (2; 4) | **<0.001** |
| L-Dopa equivalent daily dose, mg/day | 350 (229; 563) | 360 (300; 450) | 0.878 | 375 (300; 525) | 400 (0; 500) | 0.561 |
| Use of anti-hypotensive medications | 0 (0) | 0 (0) | - | 0 (0) | 3 (14) | 0.238 |
| Use of anti-hypertensive medications | 16 (47) | 38 (43) | 0.663 | 8 (47) | 5 (24) | 0.133 |
| OH | 14 (41) | 5 (6) | **<0.001** | 9 (53) | 21 (100) | **<0.001** |
| Symptoms of overactive bladder | 10 (29) | 57 (64) | **<0.001** | 13 (77) | 19 (91) | 0.378 |
| Urinary voiding difficulties | 0 (0) | 21 (24) | **<0.001** | 2 (12) | 20 (95) | **<0.001** |
| Postural instability within 2 years  from motor onset | 2 (6) | 0 (0) | 0.075 | 14 (82) | 14 (67) | 0.460 |
| Dopaminergic responsiveness |  |  | 0.074 |  |  | 0.100 |
| Poor | 0 (0) | 0 (0) |  | 11 (65) | 13 (62) |  |
| Undetermined | 6 (18) | 5 (6) |  | 2 (12) | 7 (33) |  |
| Good | 28 (82) | 84 (94) |  | 4 (24) | 1 (5) |  |
| Postural deformities | 1 (3) | 0 (0) | 0.276 | 4 (24) | 7 (33) | 0.721 |
| Pisa syndrome | 0 (0) | 0 (0) | - | 2 (12) | 4 (19) | 0.672 |
| Antecollis | 0 (0) | 0 (0) | - | 0 (0) | 4 (19) | 0.113 |
| Contractures of hands or feet | 1 (3) | 0 (0) | 0.276 | 3 (18) | 0 (0) | 0.081 |
| Bulbar dysfunction | 14 (41) | 4 (5) | **<0.001** | 12 (71) | 13 (62) | 0.575 |
| Dysarthria | 10 (29) | 2 (2) | **<0.001** | 7 (41) | 10 (48) | 0.752 |
| Dysphagia | 6 (17) | 2 (2) | 0.006 | 6 (35) | 8 (38) | 0.859 |
| Respiratory symptoms | 2 (6) | 0 (0) | 0.075 | 7 (41) | 12 (57) | 0.515 |
| Stridor | 2 (6)* | 0 (0) | 0.075 | 7 (41) | 1 (5) | 0.013 |
| Inspiratory sighs | 0 (0) | 0 (0) | - | 0 (0) | 12 (57) | **<0.001** |
| Emotional incontinence | 0 (0) | 0 (0) | - | 0 (0) | 3 (14) | 0.238 |
| 4-items MSA-P score |  |  | 0.075 |  |  | **<0.001** |
| 0 points | 16 (47) | 25 (28) |  | 0 (0) | 0 (0) |  |
| 1 point | 10 (29) | 45 (51) |  | 3 (18) | 0 (0) |  |
| 2 points | 8 (24) | 19 (21) |  | 7 (41) | 2 (10) |  |
| 3 points | 0 (0) | 0 (0) |  | 7 (41) | 6 (29) |  |
| 4 points | 0 (0) | 0 (0) |  | 0 (0) | 13 (62) |  |
| 4-items MSA-P score category |  |  | 0.794 |  |  | 0.081 |
| Low risk (i.e., 0-1 point) | 26 (77) | 70 (79) |  | 3 (18) | 0 (0) |  |
| High risk (i.e., ≥2 points) | 8 (24) | 19 (21) |  | 14 (82) | 21 (100) |  |
| 6-items MSA-P score |  |  | 0.007 |  |  | **<0.001** |
| 0 points | 14 (41) | 23 (26) |  | 0 (0) | 0 (0) |  |
| 1 point | 10 (29) | 44 (49) |  | 0 (0) | 0 (0) |  |
| 2 points | 7 (21) | 22 (25) |  | 4 (24) | 0 (0) |  |
| 3 points | 3 (9) | 0 (0) |  | 6 (35) | 2 (10) |  |
| 4 points | 0 (0) | 0 (0) |  | 6 (35) | 4 (20) |  |
| 5 points | 0 (0) | 0 (0) |  | 1 (6) | 11 (52) |  |
| 6 points | 0 (0) | 0 (0) |  | 0 (0) | 4 (19) |  |
| 6-items MSA-P score category |  |  | 0.020 |  |  | 0.032 |
| Low risk (i.e., ≤2 points) | 31 (91) | 89 (100) |  | 4 (24) | 0 (0) |  |
| High risk (i.e., ≥3 points) | 3 (9) | 0 (0) |  | 13 (77) | 21 (100) |  |

* To inquire about stridor, physicians demonstrated it to the patients and their partners with their own voice, asking if they have ever heard such sound. We acknowledge that the partners of these two Tel Aviv PD cases might have misinterpreted nocturnal stridor with snoring.

Supplementary Figure 1 – Observer-independent CHAID regression tree algorithm for the expansion of the 4-item MSA-P score


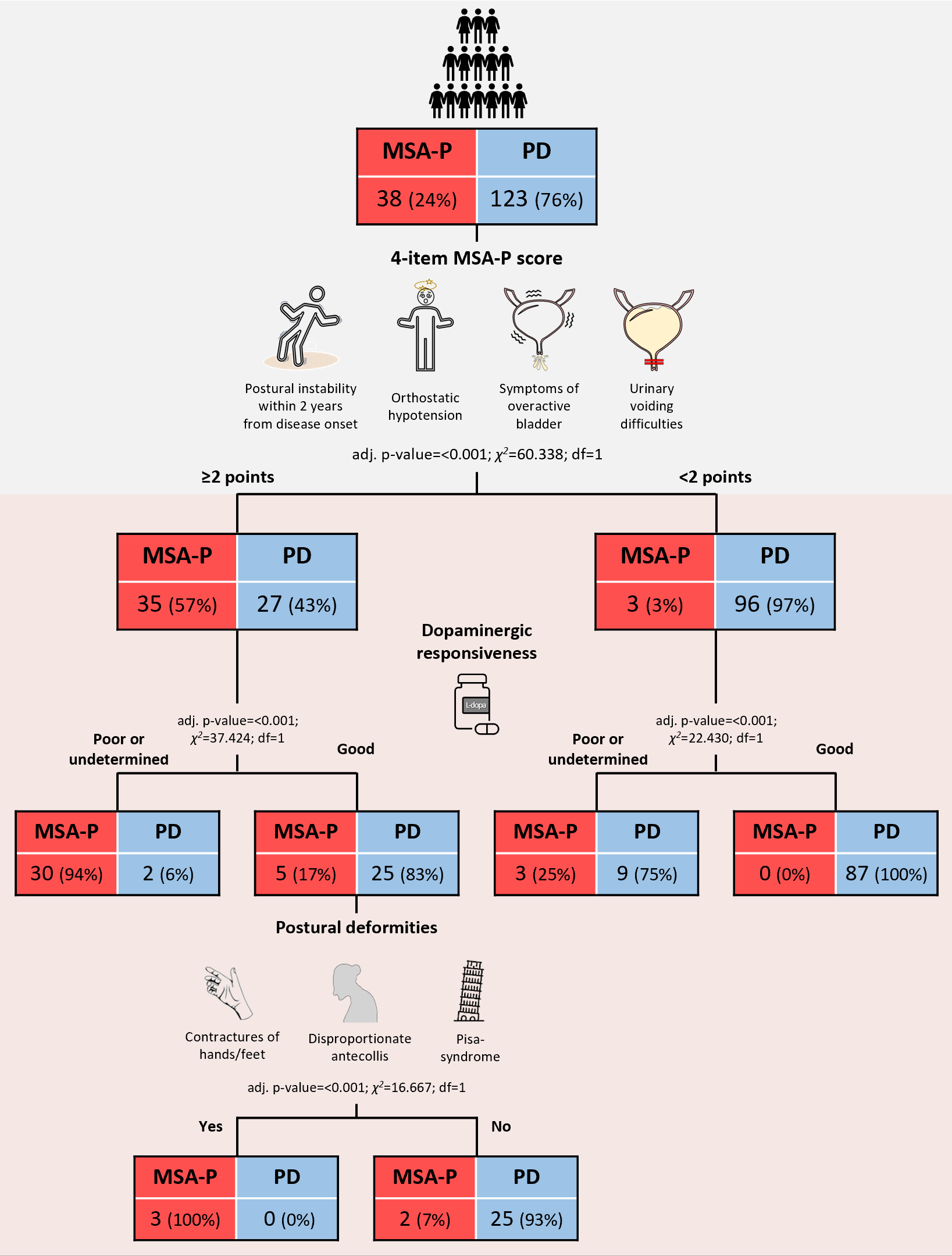
The model cross-validation was operated with a repeated automatic selection of n=10 validation samples upon removal of the first fixed node (i.e., 4-item MSA-P score low- versus high-risk category) and reproduced a decisional-tree with the same node variables shown here under, i.e. the 4-items MSA-P score, pattern of dopaminergic responsiveness and presence of postural deformities within two years from motor onset.
